# Influence of Varying Fermentation Parameters of the Yeast Strain *Cyberlindnera saturnus* on the Concentrations of Selected Flavor Components in Non-Alcoholic Beer Focusing on (*E*)-*β*-Damascenone

**DOI:** 10.3390/foods11071038

**Published:** 2022-04-02

**Authors:** Yvonne Methner, Philipp Dancker, Robin Maier, Mailen Latorre, Mathias Hutzler, Martin Zarnkow, Martin Steinhaus, Diego Libkind, Stephanie Frank, Fritz Jacob

**Affiliations:** 1 Research Center Weihenstephan for Brewing and Food Quality, Technical University of Munich, Alte Akademie 3, 85354 Freising, Germany; yvonne.methner@tum.de (Y.M.); philipp.dancker@tum.de (P.D.); m.hutzler@tum.de (M.H.); f.jacob@tum.de (F.J.); 2 Leibniz Institute for Food Systems Biology, Technical University of Munich (Leibniz-LSB@TUM), 85354 Freising, Germany; r.maier.leibniz-lsb@tum.de (R.M.); martin.steinhaus@tum.de (M.S.); s.frank.leibniz-lsb@tum.de (S.F.); 3 Centro de Referencia en Levaduras y Tecnología Cervecera (CRELTEC), Instituto Andino Patagónico de Tecnologías Biológicas y Geoambientales (IPATEC), CONICET—Universidad Nacional del Comahue, Quintral 1250, San Carlos de Bariloche CP8400, Argentina; mailenlatorre@gmail.com (M.L.); libkindfd@comahue-conicet.gob.ar (D.L.)

**Keywords:** non-*Saccharomyces* yeast, *Cyberlindnera saturnus*, fermentation, brewing, non-alcoholic beer, response surface methodology, gas chromatography-mass spectrometry, olfactometry, secondary metabolites, (*E*)-*β*-damascenone

## Abstract

The diversification of beer flavor is becoming increasingly popular, especially in the field of non-alcoholic beers, where sales are growing steadily. While flavor substances of traditional beers can largely be traced back to defined secondary metabolites, the production of non-alcoholic beers with non-*Saccharomyces* yeasts generates novel fruity flavors, some of which cannot yet be assigned to specific flavor substances. In a recently published study, besides pear, cool mint sweets, and banana-like flavor, distinctive red berry and apple flavors were perceived in a non-alcoholic beer fermented with the yeast strain *Cyberlindnera saturnus* TUM 247, whose secondary metabolites were to be elucidated in this study. The trials were carried out using response surface methodology to examine the fermentation properties of the yeast strain and to optimize the beer with maximum fruitiness but minimal off-flavors and ethanol content. It turned out that a low pitching rate, a moderate fermentation temperature, and an original gravity of 10.5 °P gave the optimal parameters. Qualitative analysis of the secondary metabolites, in addition to standard analysis for traditional beers, was first performed using headspace-gas chromatography with olfactometry. (*E*)-*β*-damascenone emerged as the decisive substance for the red berry and apple flavor and so this substance was then quantitated. Although (*E*)-*β*-damascenone is a well-known secondary metabolite in beer and this substance is associated with apple or cooked apple- and berry-like flavors, it has not yet been reported as a main flavor component in non-alcoholic beers.

## 1. Introduction

The importance of non-alcoholic beers has grown in recent years and the trend continues to grow into this direction [1,2]. The use of maltose-negative non-*Saccharomyces* yeasts is a significant production method for non-alcoholic beers to reduce or even eliminate undesirable wort off-flavors. The potential to generate pleasant flavors using the microbiological method has been shown to be effective in numerous studies. There is a particular focus on generating different fruity flavor characteristics due to a more diverse and higher ester concentration in beer [2,3,4,5,6,7,8].

Although the secondary metabolites of regular traditional beers produced with conventional *Saccharomyces* brewing yeasts have been deciphered, the use of non-*Saccharomyces* yeasts occasionally result in flavor compounds that cannot be clearly assigned in standard analysis [2,9]. A recent study by Methner et al. [9] revealed that in some non-alcoholic beers produced with various non-*Saccharomyces* yeasts, the sensory assessors perceived red berry and apple flavors that could not be directly attributed to any of the volatiles analyzed. In the specified study, one yeast strain stood out positively in terms of its flavor-generating properties in brewer’s wort: *Cyberlindnera saturnus* TUM 247. Besides a dominant cool mint sweets and red berry flavor, the beer fermented with this yeast strain was further described to exhibit less intense but perceptible pear, banana, solvent, and apple flavors. The yeast species *C. saturnus* is generally known from existing literature sources to be suitable for the production of non-alcoholic beers due to its maltose-negative properties [9,10,11]. It also synthesizes significant amounts of 3-methylbutyl acetate (isoamyl acetate) during the fermentation process in brewer’s wort resulting in a fruity, mainly banana flavor [5,10]. However, the fact that certain yeast strains of this species can also produce unusual red berry and apple flavors is a recent finding.

Apple flavor in beer is well known and can, for example, be attributed to 2-phenylethyl acetate or to ethyl decanoate. As a sour apple flavor, it can be traced back to ethyl hexanoate and ethyl octanoate. Some sources also attribute an apple flavor to isoamyl acetate. The flavor threshold concentration of 2-phenylethyl acetate in beer is 3.8 mg/L, for isoamyl acetate this is 1.2 mg/L, ethyl hexanoate 0.21 mg/L, ethyl octanoate 0.9 mg/L, and ethyl decanoate 1.5 mg/L. In addition to these five esters, acetaldehyde is also known for its apple flavor, being recognized rather as green apple and grassy when exceeding a flavor threshold concentration of 10 mg/L [6,7,8,9]. Furthermore, the ketone (*2E*)-1-(2,6,6-trimethylcyclohexa-1,3-dien-1-yl)but-2-en-1-one ((*E*)-*β*-damascenone) can also induce an apple or cooked apple-like impression in beer [12,13,14,15,16]. With regard to red berry flavor, there are currently no studies that present findings that attribute this flavor in beer to volatile substances, although berry-like flavors are principally known when using non-*Saccharomyces* yeasts for beer production [17,18]. Interestingly, however, (*E*)-*β*-damascenone has already been described in the literature as causing red fruits and strawberry flavor [19,20,21].

Therefore, this study investigated which volatile substance or substances were responsible for the red berry-like flavor expression in beer. Moreover, it was examined which volatile substances were responsible for the apple-like flavor. In addition, the question was answered as to what extent different fermentation parameters influence the flavor characteristics of the non-alcoholic beer produced with TUM 247. Studies by Pires et al. and Verstrepen et al. showed that fermentation parameters such as original gravity, temperature, and pitching rate have a demonstrable influence on the formation of esters during beer production [22,23]. Further studies by Bellut et al., Michel et al., and Puerari et al. also provided insight that the fermentation parameters have a decisive impact on the beer flavor. These three studies used response surface methodology (RSM) to optimize the fermentation parameters with regard to e.g., a maximum ester yield [10,17,24]. Based on the successful application of the method, RSM with a central composite design was used in this study to combine the variation in fermentation temperature, original gravity and pitching rate. This method was applied specifically to optimize the beer flavor and also to investigate the general influence of the different fermentation parameters on the flavor of the beers.

Since the fermentation trials were conducted in 2-L small-scale fermentation trials, sterilized wort produced from malt extract was used for this study in order to ensure a comparable, standardized wort base and avoid microbiological contamination. However, it must be taken into consideration that the thermal influence on the wort during the sterilization process generates Strecker aldehydes, resulting in the formation of various aldehydes, Maillard reaction products and ketones, such as (*E*)-*β*-damascenone [15,25,26]. Aldehydes, can cause the characteristic wort flavors and are often referred to as off-flavors [27]. However, in addition to undesirable wort off-flavors, further adverse flavor compounds, such as 3-methylbutanoic acid (isovaleric acid) or butane-2,3-dione (diacetyl), can emerge during wort fermentation, which also applies to the yeast species *C. saturnus* [9]. For this reason, these two flavor compounds were explicitly investigated. In addition to sensory evaluations by trained panelists, extensive gas chromatography-mass spectrometry (GC-MS) analyses were performed as part of this study. As a result of headspace-gas chromatography (HS-GC) with olfactometry (-O), flame ionization (-FID) and mass spectrometry (-MS) for a qualitative assessment, a GC × GC-MS system was applied for quantitation.

The main objective of this study was to find out to which flavor components the red berry and the apple flavor can be attributed in order to reconstruct which secondary metabolites were responsible for the overall fruity flavor. Additionally, the influence of different fermentation parameters was investigated by RSM to determine optimum parameters resulting in maximum fruitiness of the beer with as few off-flavor compounds as possible. At the same time, the ethanol content was to be kept as low as possible, since the target application was a non-alcoholic beer.

## 2. Materials and Methods

### 2.1. Yeast Strain and Wort

The maltose-negative yeast strain *Cyberlindnera saturnus* TUM 247 proved suitable for producing non-alcoholic beers in preliminary trials based on its fermentation properties. The favorable fermentation properties were due to the fact that only glucose, fructose, and sucrose could be metabolized from brewer’s wort, while exhibiting sufficient hop tolerance and producing an exceptionally pleasant fruity flavor [9,18]. The yeast strain was isolated from soil underneath an ash tree (*Fraxinus excelsior*) in the district Kelheim, state Bavaria, Germany. Identification was performed by D1/D2 26S rDNA sequencing using primers according to Kurtzman [28] fp NL1 rp NL4, while NL1 was used as sequencing primer. The 26S sequence data from sequencing with NL1 primer can be found in the supplementary material Sequence S1.

For the RSM experiments, the wort was prepared from unhopped malt extract (Weyermann^®^, Bamberg, Germany), which was adjusted to the different, desired original gravities. The wort was then sterilized (100 °C, 45 min) to exclude microbiological contamination.

Furthermore, to determine the extent to which (*E*)-*β*-damascenone can be formed via thermal processing, three different thermally treated worts were prepared from unhopped malt extract (Weyermann^®^, Bamberg, Germany). The worts were all adjusted to an original gravity of 7 °P and 1800 mL of each was filled into 2000 mL Duran glass bottles (Schott AG, Mainz, Germany). While one wort was kept at 100 °C for 5 min (pH 5.6), another wort was sterilized at 100 °C for 45 min (pH 5.5). The third wort was autoclaved at 121 °C for 10 min (pH 5.4). The wort with the lowest thermal input was used for small-scale fermentation trials as described in Methner et al. (2022) [9].

### 2.2. Yeast Propagation and Fermentation

For propagation, the yeast strain TUM 247 was inoculated from wort slope agar under sterile conditions into five 500 mL flasks each containing 250 mL of unhopped wort (9.0 °P, pH 5.4). After propagating for 72 h at 20 °C on a WiseShake orbital shaker (Witeg Labortechnik GmbH, Wertheim, Germany) at 80 rpm, the yeast suspensions were transferred to five sterile 2500 mL flasks, each containing 1800 mL of comparable wort. After a further propagation period of 60 h and 12 h of settling time, the wort supernatant was poured off, accounting for approximately one third of the total volume, before yeast cell numbers were counted using the Cellometer^®^ Vision (Nexcelom Bioscience LLC, Lawrence, MA, USA). Subsequently, the required amounts of propagation yeast could be calculated for the fermentation trials.

The wort for the fermentation trials was prepared analogously to the propagation wort. Before pitching, the propagation yeasts were centrifuged (Roto Super 40, Andreas Hettich GmbH & Co. KG, Tuttlingen, Germany) in sterilized 500 mL PPCO centrifuge bottles (Nalgene, Thermo Fisher Scientific, Waltham, MA, USA) at 750× *g* for 5 min and the supernatant was discarded. The yeasts were then washed in sterile physiological sodium chloride solutions before being used for fermentation. In total, 20 small-scale fermentation trials and an optimization trial in triplicate were conducted, each unpressurized in 1800 mL sterilized unhopped wort in 2000 mL Duran glass bottles (Schott AG, Mainz, Germany) equipped with fermentation locks on top. After fermentation for seven days (168 h), the fermentation locks were replaced with sterile screw caps and the 20 samples were cooled down to 3 °C. Following storage for 72 h, the samples were analyzed and tasted.

### 2.3. Response Surface Methodology (RSM)

To investigate the influence of different fermentation parameters on selected flavor components and ethanol content of the beers fermented with the yeast strain *C. saturnus* TUM 247, RSM was applied using Design-Expert 13 software (StatEase, Minneapolis, MN, USA). A face-centered central composite design with three independent factors and six replications of the center point was chosen. The three variable factors were original gravity (7 to 11 °P), fermentation temperature (12 to 28 °C), and pitching rate (5 × 10^6^ to 25 × 10^6^ cells/mL). Therefore, in total, 20 small-scale fermentation trials were performed. This results in a center point with the factors of temperature at 20 °C, original gravity of 9 °P, and pitching rate of 15 × 10^6^ cells/mL. Table 1 presents the experimental design.

All data were statistically evaluated with analysis of variance (ANOVA). A one-sample *t*-test was performed using OriginPro 2020 as statistical software to evaluate whether all values of the six center points were insignificantly different from each other. If there was no significant difference in the one-sample *t*-test, it may be assumed that all remaining 14 measurement points reveal statistically significant results.

As responses, concentrations of ethanol, (*E*)-*β*-damascenone, the sum of esters—isoamyl acetate in particular—isovaleric acid, diacetyl, the fruitiness of the beer, and the sum of DLG points were taken into account. For optimization using RSM, the aim was to maximize the concentrations of (*E*)-*β*-damascenone, the sum of esters, isoamyl acetate, the fruitiness of the beer, and the sum of DLG points, while minimizing the concentrations of ethanol, isovaleric acid and diacetyl. Only the responses to be optimized were analyzed in the beer produced in the course of the RSM optimization.

### 2.4. Analytical Methods

Regarding the analytical methods, MEBAK ^1^ standard methods for wort and beer were initially applied for the 20 beer samples (cf. Table 1) and additionally in the final stages for the optimized beer samples. Since the standard methods were not sufficient to elucidate the red berry- and apple-like flavors of the beers, qualitative HS-GC-O, -FID, and -MS were performed first, followed by a stable isotope dilution assay (SIDA) using a GC × GC-MS system for quantitation.

#### 2.4.1. Analytical Standard Methods

The analytical standard methods applied for the initial wort and the beers are listed in Table 2. 

#### 2.4.2. Headspace-Gas Chromatography (HS-GC) with Olfactometry (-O), Flame Ionization (-FID) und Mass Spectrometry (-MS)

For this method, a multidimensional GC system consisting of two GC-2010Plus (Shimadzu, Neufahrn bei Freising, Germany) connected by a Deans’ switching device was used. GC-1 was equipped with a polar fused silica capillary ZB-WAX (30 m × 0.25 mm × 0.25 µm, Phenomenex Ltd., Aschaffenburg, Germany) and GC-2 with a non-polar fused silica capillary column ZB-5MS (30 m × 0.32 mm × 0.5 µm, Phenomenex Ltd., Aschaffenburg, Germany). As a static headspace sampler and injection port, the HS-20 series (Shimadzu, Neufahrn bei Freising, Germany) was used. After equilibration of the 10 mL beer samples, each in 20 mL HS vials, a portion of the HS was transferred, splitless, for 1 min onto the GC column using a 1 mL sample loop. The conditions for the HS-20 are specified in the supplementary material Table S1. The GC oven temperature was started at 40 °C to improve the separation of early eluting components. After that, the temperature was increased in 10 °C/min increments up to 125 °C and in 20 °C/min increments up to 240 °C and held there for 5 min. This equals a total runtime of 19.75 min. The temperature program was the same for both GCs and the inlet pressure was set for an optimal carrier gas velocity for helium between 20–40 cm/s. After elution on GC-1 the components were split between either the FI-/O-Detector on GC-1 or transferred onto the GC-2 for further separation and to reduce overlays. The partition between the FI-/O-Detector were achieved via a SGE SilFlow GC 4 Port Splitter (Trajan Scientific Pty Ltd., Ringwood, VIC, Australia) in a 1:1 ratio. On the GC-2, the eluting components were analyzed by a mass spectrometer QP2010-Ultra SE (Shimadzu, Neufahrn bei Freising, Germany) with electron ionization (EI) at 70 eV. The interface I/F was set at 250 °C and the ion source at 200 °C while the scan was between 50–250 amu. The emission current was at 60 µA.

Using GC-O, the odor qualities of the odorants in the beer samples in Runs 1–20 with the fermentation parameters shown in Table 1 were analyzed for the specific berry and apple flavor. For the final identification, the odor quality, retention index (RI), and mass spectrum was compared with that of the corresponding reference compound. The linear RI was calculated from its retention time and the retention times of adjacent *n*-alkanes by linear interpolation [29].

#### 2.4.3. GC × GC-MS System

A 6890 Plus gas chromatograph (Agilent Technologies, Waldbronn, Germany) was equipped with a Combi PAL autosampler (CTC Analytics, Zwingen, Switzerland), a KAS4 injector (Gerstel, Mülheim/Ruhr, Germany), and a fused silica column, DB-FFAP, 30 m × 0.25 mm i.d., 0.25 μm film (Agilent Technologies, Waldbronn, Germany). The carrier gas was helium at 2.0 mL/min constant flow. The end of the column was connected to a second fused silica column, DB-5, 2 m × 0.15 mm i.d., 0.30 μm film (Agilent Technologies, Waldbronn, Germany). The front part of this column passed through a liquid nitrogen-cooled dual stage quad-jet thermal modulator (Leco, Mönchengladbach, Germany). The modulator was used to collect the volatiles eluting from the first column in discrete portions (4 s), which were then rechromatographed on the major part of the second column. This part was installed in a secondary oven mounted inside the primary oven of the gas chromatograph. The end of this column was connected via a heated (250 °C) transfer line to the inlet of a Pegasus III time-of-flight (TOF) mass spectrometer (Leco, Mönchengladbach, Germany). The temperature of the first oven was 40 °C for 2 min, followed by a gradient of 6 °C/min to a final temperature of 230 °C, which was held for 5 min. The temperature of the secondary oven was 80 °C for 2 min, followed by a gradient of 6 °C/min to a final temperature of 250 °C, which was held for 5 min. Mass spectra were generated in the EI mode at 70 eV, a scan range of *m*/*z* 35−300, and a scan rate of 100 spectra/s. The ChromSpace software (Markes International Ltd., Llantrisant, UK) was employed for data analysis.

(*E*)-*β*-damascenone was a gift from Symrise to the Leibniz-LSB@TUM (Holzminden, Germany) and (^2^H_4−7_)-(*E*)-*β*-damascenone was synthesized as detailed in the literature [30]. Diethyl ether (VWR, Darmstadt, Germany) was freshly distilled before use. For the work-up, beer was filtered through a paper filter to remove carbon dioxide. For the quantitation of (*E*)-*β*-damascenone, diethyl ether (100 mL) and (^2^H_4−7_)-(*E*)-*β*-damascenone (0.013 μg) were added to the samples (50 mL). The mixture was stirred for 30 min at ambient temperature. The organic phase was separated, and the aqueous phase was shaken with a second portion of diethyl ether (100 mL). The combined organic phases were washed with saturated aqueous sodium chloride solution (2 × 50 mL) and dried over anhydrous sodium sulfate. Nonvolatiles were removed by solvent-assisted flavor evaporation (SAFE) [31] at 40 °C. The distillate was concentrated to 500 μL by using a Vigreux column (50 × 1 cm) and a Bemelmans microdistillation device [32]. The concentrate was analyzed using the GC × GC-MS system. Peak volumes of the (*E*)-*β*-damascenone peak and the (^2^H_4−7_)-(*E*)-*β*-damascenone peak were extracted using ions *m*/*z* 121 for (*E*)-*β*-damascenone and *m*/*z* 125−128 for (^2^H_4−7_)-(*E*)-*β*-damascenone. The concentration of (*E*)-*β*-damascenone in the samples was then calculated from the volume of the (*E*)-*β*-damascenone peak, the volume of the (^2^H_4−7_)-(*E*)-*β*-damascenone peak, the amount of sample used, and the amount of (^2^H_4−7_)-(*E*)-*β*-damascenone added, by employing a calibration line equation. To obtain the calibration line equation, diethyl ether solutions of (*E*)-*β*-damascenone and (^2^H_4−7_)-(*E*)-*β*-damascenone mixtures in different concentration ratios were analyzed under the same conditions followed by linear regression. The resulting calibration line equation was y = 0.9608x + 0.1701, with y = [concentration of (^2^H_4−7_)-(*E*)-*β*-damascenone]/[concentration of (*E*)-*β*-damascenone] and x = [peak volume of (^2^H_4−7_)-(*E*)-*β*-damascenone]/[peak volume of (*E*)-*β*-damascenone].

### 2.5. Sensory Evaluation

The tastings were performed by twelve DLG-certified (Deutsche Landwirtschafts-Gesellschaft e.V., Frankfurt, Germany) assessors in a specially designated room (neutral white-colored with individual tasting chambers) at 20 °C. The samples were tempered to 12 °C before tasting and were assigned three-digit randomized numbers. First, the tasters performed an evaluation according to the DLG scheme, which comprises a rating scale from zero (minimum score) to five (maximum score) [33], to evaluate only the odor, the purity of taste and the body of the beers. Since the beers were unhopped and fermented without pressure, the quality of the bitterness and carbonation were not taken into account in the sensory evaluation. By conducting the DLG test, beers were, therefore, mainly evaluated for their purity in terms of odor and taste, so that a devaluation was based on perceptible off-flavors such as a buttery flavor from diacetyl or vegetable-like flavor from dimethyl sulfide.

The tasters then evaluated the fruitiness of the beers. In this context, a scale of zero to five was used to indicate how fruity the beer was rated, with zero standing for not at all fruity and five for maximum fruitiness. Due to the large number of samples, a composite sample was prepared from the six center points, each in equal proportions so that the assessors evaluated a total of 15 samples. Prior to this, as described in Section 2.3. the six center point samples were analyzed and statistically evaluated using a one-sample *t*-test to ensure that the six samples did not differ significantly. The fruitiness was converted to a percentage based on a maximum score of 60 points for the evaluation. Since the flavor profile of the yeast strain *C. saturnus* TUM 247 is already known from a recent study [9] and is described as being like cool mint sweets and solvent-like besides fruity expressions of pear, banana, red berries, and apple, the tasters were specifically asked if they could perceive these flavors in the beers. Additionally, based on the analyzed secondary metabolites, the flavor characteristics honey, cheesy, and watery/pale were included. The methods for sensory evaluation were applied both to the 15 beer samples from the RSM central composite design and to the beer sample optimized by RSM in triplicate.

To establish a possible correlation between the specific volatile compounds measured analytically and the specified flavor characteristics identified by the tasters, a principal component analysis (PCA) was performed after normalizing the values using OriginPro 2020 statistical software.

## 3. Results

In this chapter, the analyses of the 20 beer samples (cf. Table 1) are presented, which were carried out according to the experimental design of the RSM (cf. Section 2.3). This includes standard analytical methods for beer (cf. Table 2) and two additional GC methods (Section 2.4). Furthermore, the sensory evaluation of the fermented samples is depicted before the RSM optimization results are presented. The yeast strain TUM 247 (cf. Section 2.1) was used for all experiments without exception using the propagation and fermentation methods described in Section 2.2.

### 3.1. Analytical Results

Initially, the one-sample *t*-test was performed for selected values. Since the mean population was insignificantly different from the test mean at the 0.05 level without exception (cf. supplementary material Table S2), it may be assumed that all remaining 14 measurement points show statistically significant results in a single experiment.

In Table 3, an analysis of variance (ANOVA) was performed for the purposes of RSM for predetermined responses (cf. Section 2.3) that were later used for the optimization experiment. Occasionally, values were identified as outliers in the statistical analyses. Thus, for the sum of esters and isoamyl acetate, Run 16 was excluded; for diacetyl, Run 2; for (*E*)-*β*-damascenone, Run 13; and for fruitiness, Run 12. Additionally, for (*E*)-*β*-damascenone Runs 7, 8, 10, 19, and 20 were excluded, which is explained in the further course.

All eight responses revealed significant models. Although some responses marked with asterisks* showed a significant lack of fit (LOF), these were included in the analysis due to the significant *p*-values. However, only three-dimensional (3D) plots were created for significant models with insignificant LOF.

In addition to the responses to be optimized in the RSM, the results of the standard analytical methods from Section 2.4.1 are depicted below. The analytical results of ethanol, apparent attenuation, and pH values of the 20 samples depicted in Table 4 showed correlations depending on the selected fermentation parameters.

Regarding the ethanol content, the general trend was that the higher the original gravity and the temperature, the higher the ethanol content. In case a lower pitching rate was used, correspondingly less ethanol was produced by the yeasts. Conversely, the higher the pitching rate, the higher the ethanol content. With the exception of Run 13, all beers were below 0.5% (*v*/*v*) ethanol content. The low ethanol contents can be explained by the fact that the yeast species *C. saturnus* is known to be maltose- and maltotriose-negative [11].

The situation was similar for the apparent attenuation. Since the apparent attenuation and the ethanol content are directly related, this behavior was to be expected. The lower the original gravity, the fewer sugars are available for the yeast, so that correspondingly fewer sugars can be converted into ethanol and carbon dioxide. It is interesting to note that at a fermentation temperature of 12 °C, the yeast activity was consistently weak, irrespective of the original gravity. Only with increasing fermentation temperatures was there an increase in the yeast metabolizing the available wort sugars. The strongest activity within the selected temperature range was at the maximum of 28 °C.

Evaluating the pH values of the beers, a linear correlation can be assumed between the original gravity and the temperature. A lower yeast pitching rate resulted in a higher pH value, while the pH value decreased as the pitching rate increased. The stronger pH drop at higher fermentation temperatures can be attributed to the fact that the yeasts form more fixed and volatile organic acids due to their higher fermentation activity [34]. Nevertheless, it could be assumed that the pH drop would be correspondingly stronger at an original gravity of 11 °P than at 7 °P due to the higher substrate concentration, which was not the case. The yeast species *C. saturnus*, as already mentioned, can only metabolize glucose, fructose, and sucrose. Since these three sugars are only present in small amounts in the wort (7–9% hexoses, 3% sucrose [34]), the pH drop was relatively weak compared to a conventional brewing yeast such as *Saccharomyces cerevisiae*, which causes a pH drop in the beer to 4.2–4.6 [35].

**Figure 1:**
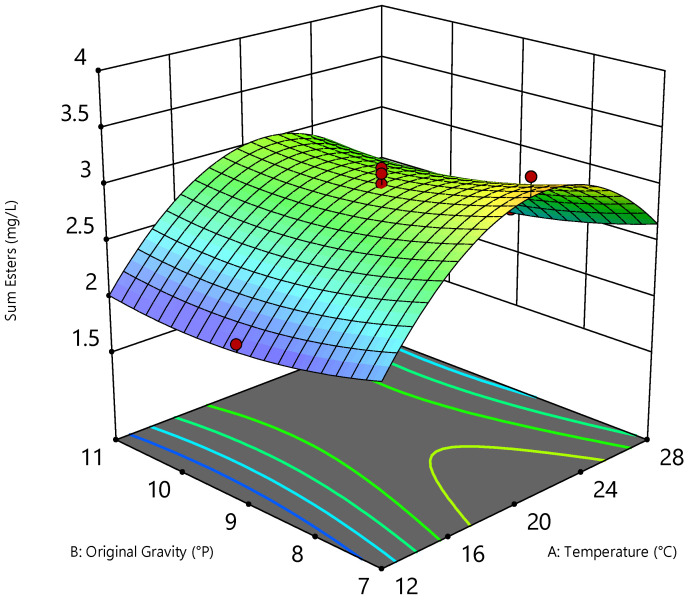
Three-dimensional (3D) diagram of the quadratic correlation between original gravity (°P), temperature (°C), and the sum of esters concentration (mg/L) of the beers at a pitching rate of 15 × 10^6^ cells/mL.

As Table 5 depicts, out of the ten quantitated fruit esters, ethyl acetate, isoamyl acetate, 2-phenylethyl acetate, ethyl hexanoate, and, to some extent, isobutyl acetate, ethyl butanoate, and ethyl octanoate were formed in concentrations above the limit of quantitation. Considering the relevant seven fruit esters analyzed in Figure 1, the 3D diagram illustrates the relationship between original gravity, temperature and the sum of esters concentration at a pitching rate of 15 × 10^6^ cells/mL.

It is particularly interesting that the concentration of the sum of esters was only slightly dependent on the original gravity, as it showed an increasing tendency with decreasing original gravity, which would not have been expected based on existing literature. The higher the original gravity, the higher the concentration of nitrogen, fermentable sugars, and unsaturated fatty acids. These are required for ester synthesis, since fusel alcohols are formed via nitrogen metabolism and acetyl-CoA via sugar and lipid metabolism, which, in turn, react via ester synthase to form fatty acid esters. Accordingly, with increasing original gravity, more substrate is available for ester synthesis [23,34]. *C. saturnus* TUM 247 exhibited a different metabolism to conventional brewing yeasts, since the investigations showed that the original gravity had almost no influence on ester formation. There was even a tendency for more esters to be metabolized at lower original gravity. Thus, the highest concentration of the sum of esters was measured at 7 °P original gravity and at a pitching rate of 5 × 10^6^ cells/mL (cf. Table 5), which was not to be expected as the literature explains that higher pitching rates often lead to higher ester concentrations [34,36,37]. Up to a pitching rate of approximately 18 × 10^6^ cells/mL, the concentration of the sum of esters decreased continuously and independently of temperature. Regardless of the pitching rate and the original gravity, the ester peak was always between 20 and 22 °C. Up to 22 × 10^6^ cells/mL the total ester concentration increased slightly in the higher investigated original gravity concentrations, so that from these cell numbers the ester concentrations were almost independent of the selected original gravity range between 7 and 11 °P. Above 22 × 10^6^ cells/mL, the total esters in the beers increased again, although at the maximum applied cell count of 25 × 10^6^ cells/mL and 7 °P, this was still approximately 1.5 mg/L lower than at 5 × 10^6^ cells/mL at unchanged temperature as well as original gravity. Nevertheless, a similar observation was made in a study by Bellut et al. [10] in which a *Cyberlindnera subsufficiens* also exhibited enhanced fruitiness in beer at a comparatively low pitching rate. However, this was at a pitching rate of 1 × 10^7^ cells/mL. Measured on the basis of regular beers containing alcohol, only the isoamyl acetate concentrations were in the range of the flavor threshold concentration, which is at approximately 1.2 mg/L, causing fruity, banana, pear, and also solvent-like flavors [14,38,39,40]. Nevertheless, due to the low alcohol concentrations and the associated low concentrations of higher alcohols, it could be assumed that the flavor threshold concentrations are significantly lower for these kinds of beers. Accordingly, a solvent-like, fruity and sweet flavor could occur due to the presence of ethyl acetate, whereas 2-phenylethyl acetate leads to floral, honey, apple-like flavor impressions [41,42]. While ethyl acetate is increasingly formed by the yeasts at higher fermentation temperatures, the pitching rate had a significant influence as well. For the selected range, it could be determined that the higher the pitching rate, the higher the ethyl acetate concentration in the beer. Interestingly, the opposite conclusion could be drawn for isoamyl acetate with regard to the pitching rate. In addition, a lower original gravity resulted in higher isoamyl acetate concentrations, and, just as with ethyl acetate, higher temperatures also led to increased formation of the ester. 2-phenylethyl acetate was formed most strongly by the yeast at a fermentation temperature of 20 °C and decreased with both increasing and decreasing fermentation temperatures. The original gravity only played a subordinate role, since only a slightly higher 2-phenylethyl acetate formation was recorded at lower original gravity than at higher concentrations. Similar to the isoamyl acetate formation, a lower pitching rate led to higher 2-phenylethyl acetate concentrations in the beers. It is noticeable that the trend continued between pitching rates of approximately 5 to 20 × 10^6^ cells/mL. Above 20 × 10^6^ cells/mL, the flavor compound concentration increased again up to the observation limit of 25 × 10^6^ cells/mL.

**Figure 2:**
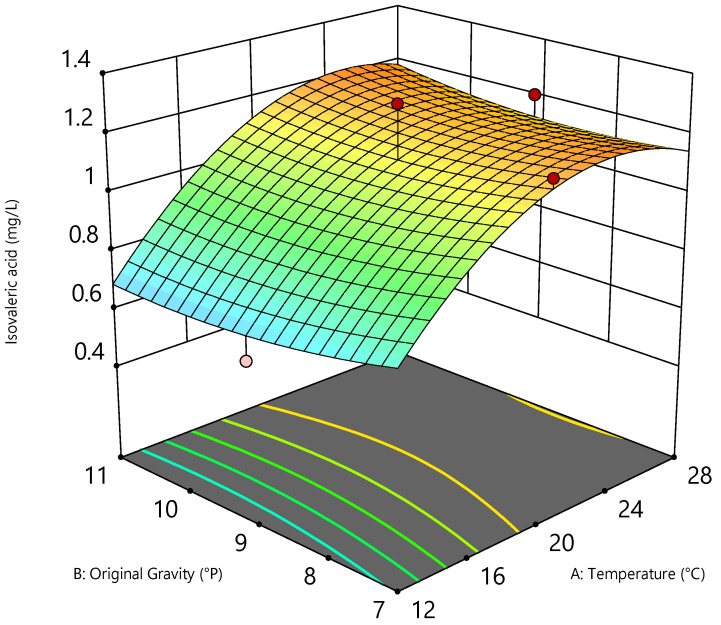
Three-dimensional (3D) diagram of the quadratic correlation of original gravity (°P), temperature (°C), and isovaleric acid concentration (mg/L) of the beers at a pitching rate of 15 × 10^6^ cells/mL.

Looking at the sum of the organic acids analyzed, which are composed of isovaleric, hexanoic, octanoic, and decanoic acid, Table 5 shows that, apart from isovaleric acid, all other quantitated acids were below the limit of quantitation and, thus, seemed not to effect the beers. However, the isovaleric acid concentration varied among the beers. Figure 2 shows the quadratic correlation of the original gravity and fermentation temperature in the isovaleric acid concentration of the beers at a pitching rate set at 15 × 10^6^ cells/mL.

It was not possible to ascertain a dependence of the isovaleric acid concentration on the original gravity. However, the fermentation temperature and the pitching rate played a significant role. Rising temperatures between 12 °C and 24 °C led to an increase in isovaleric acid production by the yeast. A plateau can be seen at fermentation temperatures between 24 °C and 28 °C. The lower the selected pitching rate, the less isovaleric acid was formed (cf. Table 5). However, at a pitching rate of 15 × 10^6^ cells/mL, the concentration did not increase further in the beers. One possible reason for this could be that certain amino acids were depleted from the wort and were, therefore, no longer available for yeast metabolism, as isovaleric acid is a by-product of the amino acid metabolism formed from leucine during catabolic pathway [43]. In regular beers containing ethanol, the flavor threshold concentration of isovaleric acid is at 1.5–2.5 mg/L, which was not measured in any of the beer samples. The maximum concentration was 1.3 mg/L. In addition, it is known from literature that isoamyl acetate has a masking effect on isovaleric acid and, therefore, based on the measured values, it could be assumed that isovaleric acid had no influence on the beers by exposing a cheesy or rancid flavor impression [38,44].

**Figure 3:**
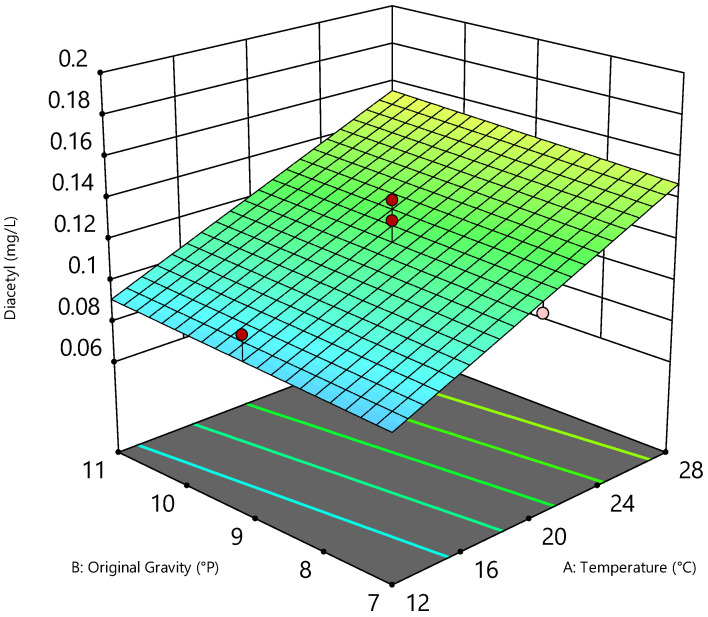
Three-dimensional (3D) diagram of the linear correlation of original gravity (°P), temperature (°C), and diacetyl concentration (mg/L) of the beers at a pitching rate of 15 × 10^6^ cells/mL.

Higher alcohols were present in lower concentrations at lower fermentation temperatures, regardless of the original gravity, and increased linearly with increasing temperature as well as increasing pitching rate (cf. Table 5). Acetaldehyde and diacetyl in the beers behaved similarly. While acetaldehyde in regular beers leads to fruity, green apple, grassy flavors only at concentrations above 10 mg/L, the flavor threshold concentration for diacetyl is 0.10 to 0.15 mg/L, causing a butter-like flavor [20,38]. The maximum value of acetaldehyde was 2.1 mg/L (cf. Table 5), which is far below the flavor threshold concentration. The situation was different for diacetyl. With values of up to 0.19 mg/L, diacetyl reached concentrations above the flavor threshold concentration. The diacetyl concentration of Run 2 at 0.29 mg/L was excluded as it was an outlier based on statistical analysis (cf. Table 5). Nevertheless, a sensory input could not be excluded and was investigated in the further course of the study. Looking at the 3D diagram in Figure 3, there is a visible linear correlation between the original gravity and the fermentation temperature on the diacetyl concentration.

The lower the selected pitching rate, the lower the measurable diacetyl concentration in the beers (cf. Table 5). While the influence of the original gravity was negligible, the fermentation temperature had a decisive influence. The 3D diagram clearly depicts that significantly less diacetyl was formed at a fermentation temperature of 12 °C than at 28 °C. The reason for this is that higher fermentation temperatures lead to stronger yeast growth, which, in turn, results in initially higher diacetyl production rates. Although diacetyl would be degraded more rapidly again at higher temperatures in the course of maturation, the short fermentation and maturation time of only seven days was not sufficient for this [45]. Moreover, the pH value was not optimal for the fastest possible diacetyl degradation, as the beers had relatively high pH values (cf. Table 4). According to the literature, the optimum pH value for acetoin dehydrogenase is 3.5 and this decreases steadily with increasing pH values [46]. Thus, the diacetyl concentration increases linearly with increasing temperature and pitching rate.

HS-GC-O, -FID, and -MS were performed on the beers listed in Table 1 to determine which flavor compound beyond standard analysis (cf. Table 2) was causing the red berry- and apple-like flavors in the beers. (*E*)-*β*-damascenone was clearly perceptible as red fruits and apple in olfactometry and was perceived as very pleasant. Using this method, it was possible to identify the odor-active compound qualitatively, although not quantitatively. Therefore, in a further step, (*E*)-*β*-damascenone was quantitated by SIDA using a GC × GC-MS system. The results are depicted in Table 6. As there was no significant difference between the six center point samples based on the one-sample *t*-test, only Run 3 was analyzed as the center point. This was the reason why Runs 7, 8, 10, 19, and 20 were excluded for ANOVA in the context of the RSM.

**Figure 4:**
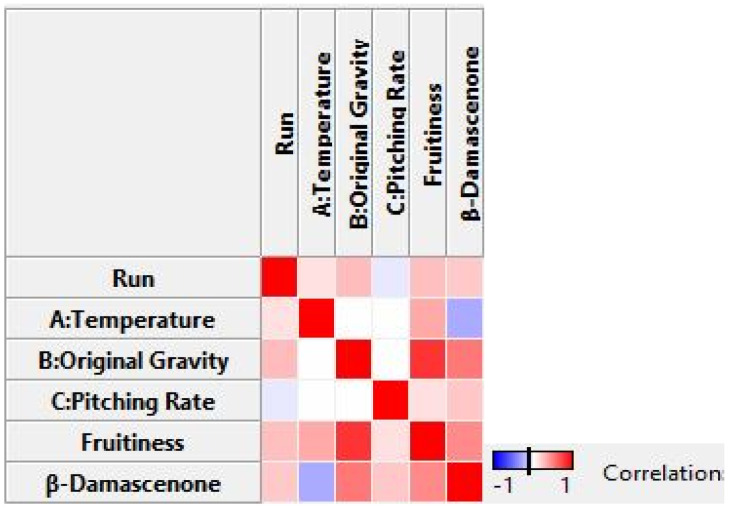
Visualization of Pearson’s correlation of selected responses (fruitiness and (*E*)-*β*-damascenone) and factors A, Temperature; B, Original Gravity; and C, Pitching Rate of the response surface methodology (RSM) for the determination of linear correlations between two variables where 1 stands for a strong positive correlation, 0 for no correlation, and −1 for a strong negative correlation.

A general trend is visible that the higher the pitching rate, the higher the (*E*)-*β*-damascenone concentrations. The fermentation temperature played more of a subordinate role, with lower temperatures tending to result in somewhat higher concentrations than higher temperatures. The original gravity, on the other hand, had a significant influence on the (*E*)-*β*-damascenone concentration, as the higher it was, the higher the measured concentrations. These conclusions can only be drawn when Run 13 is considered an outlier. Although the general variation is small, as can be seen Table 6, the *p*-value (<0.0058) for (*E*)-*β*-damascenone (cf. Table 3) showed that the values were significant. As the linear model showed a significant LOF, no 3D plot was created. Instead, a Pearson’s correlation was created to determine the linear correlation between two variables, which is depicted in Figure 4.

Pearson’s correlation confirmed that the pitching rate was positively correlating with the (*E*)-*β*-damascenone concentration and that there was an even stronger positive correlation between the original gravity and the (*E*)-*β*-damascenone concentration. The fruitiness of the beers also showed a decisive positive correlation with the (*E*)-*β*-damascenone concentration. The raw data can be found in the supplementary material Figure S2.

Based on existing literature sources, it would have been assumed that (*E*)-*β*-damascenone originated from precursors in the malt and was formed during and after the wort boiling process [47]. Therefore, as described in Section 2.1, three different thermally treated worts were analyzed for (*E*)-*β*-damascenone. In the cooked wort (100 °C for 5 min), the (*E*)-*β*-damascenone concentration stayed below the limit of quantitation. The sterilized wort (100 °C for 45 min) exhibited a (*E*)-*β*-damascenone concentration of 0.634 µg/L on average so that the thermal influence is visible. In a direct comparison to the sterilized wort, a mean value of 1.01 µg/L was measured in the five beers from the RSM with an original gravity of 7 °P. Accordingly, besides the thermal influence on the (*E*)-*β*-damascenone formation in the wort, it could be assumed that the yeast strain TUM 247 formed about 0.4 µg/L (*E*)-*β*-damascenone during fermentation. The thermal influence of the (*E*)-*β*-damascenone formation becomes even clearer by considering the autoclaved wort (121 °C for 10 min). A comparatively high concentration was determined at an average of 2.15 µg/L. To determine if (*E*)-*β*-damascenone could actually be formed by the yeast strain during fermentation, an additional triplicate fermentation experiment was performed based on the cooked wort with the lowest thermal impact. Both the initial wort and the experimental beers were analyzed by SIDA using a GC × GC-MS system. While ≤0.1 µg/L (*E*)-*β*-damascenone was detected in the wort, a concentration between 0.451 and 0.571 µg/L was measured in the three beers. It is, therefore, reasonable to assume that the yeast strain TUM 247 was able to form (*E*)-*β*-damascenone during fermentation.

### 3.2. Sensory Evaluation Results

As described in Section 2.5, due to the large number of samples, a composite sample was prepared from the six center points, each in equal proportions. This was statistically possible as, using a one-sample *t*-test at α = 0.05, there were non-significant differences in the center point samples. Table 7 shows the sum of the DLG ratings as well as the fruitiness in percent (n = 12) of the 15 beers fermented with different parameters. As the LOF of the DLG ratings and fruitiness was insignificant within the scope of the RSM (cf. Table 3), no 3D diagrams were created.

Regarding the sum of DLG points, at a pitching rate of 15 × 10^6^ cells/mL, the fermentation temperature had more of a subordinate influence, while the sum of DLG points increased with increasing original gravity up to 11 °P and was decidedly good with around 4.7 out of a total of 5.0 possible points. By reducing the pitching rate, the DLG sums decreased, while with an increasing pitching rate the DLG sums continued to rise, especially in the lower original gravity ranges between 7 and 9 °P. As off-flavors in beer lead to a devaluation in the DLG evaluation, it can be assumed, based on the results, that diacetyl had no negative influence. Diacetyl concentrations above the flavor threshold concentration were measured, especially at high fermentation temperatures and high pitching rates (cf. Table 5 and Figure 3). Nevertheless, the beers with high pitching rates, in particular, received the best DLG ratings, while the fermentation temperature had hardly any influence. Thus, neither diacetyl could be perceived as a buttery off-flavor, nor was found to be pleasant in the beers, without exception. Isovaleric acid also did not seem to affect the beer flavor negatively, as the beers with higher isovaleric acid concentrations were rated very good in the DLG testing. In terms of the fruitiness of the beers, the selected fermentation temperatures between 12 and 28 °C had little influence. Only a slight drop in fruitiness could be observed at lower temperatures. As with the sum of DLG points, however, the original gravity played a decisive role, and the fruitiness increased linearly from 7 °P to 11 °P. A change in the pitching rate did not cause any significant alteration in fruitiness.

**Figure 5:**
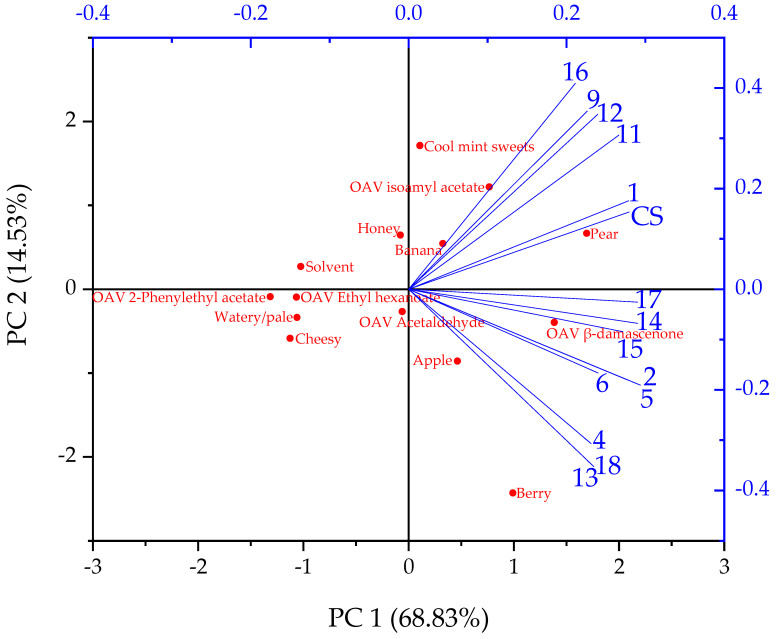
Principal Component Analysis (PCA) biplot with two principal components PC 1 and PC 2 showing the correlation of the 15 beers produced with the yeast strain C. saturnus TUM 247 as loading plot and the flavor characteristics described by the sensory assessors as well as odor activity values (OAV) of (*E*)-*β*-damascenone, isoamyl acetate, ethyl hexanoate, 2-phenylethyl acetate, and acetaldehyde.

Considering the results of the descriptive tasting with predetermined flavor attributes (raw data can be found in the supplementary material Table S3), the correlations with the 15 beers can be seen in Figure 5 as a Principal Component Analysis (PCA) plot. The odor activity values (OAV) of (*E*)-*β*-damascenone, isoamyl acetate, ethyl hexanoate, 2-phenylethyl acetate, and acetaldehyde were included in the statistical analysis to determine whether there was a direct correlation between the flavor attributes identified during the sensory evaluation and the five analyzed secondary metabolites. As mentioned in the introduction, (*E*)-*β*-damascenone, isoamyl acetate, ethyl hexanoate, 2-phenylethyl acetate, and acetaldehyde are possibly known to be responsible for apple-like flavors. Although ethyl octanoate and ethyl decanoate can also refer to apple-like flavors, their OAVs < 1 are unlikely to cause a noticeable effect on the beer flavor in comparison to the other five flavor compounds. The OAVs are depicted in Table 8. They are calculated as the ratio of the concentration to the orthonasal odor threshold concentration in water.

Principal Components (PC) 1 and 2 cover 83.36% of the variation in the data, which represents a high share. The flavor attributes cheesy, solvent, and watery/pale have no correlation with the beers, so they are not significant. Since isovaleric acid contributes to a cheesy flavor it can be assumed that this substance did not affect the flavor of the beers. Similarly, the OAVs of 2-phenylethyl acetate as well as ethyl hexanoate showed no direct correlation with the evaluated flavor attributes of the beers. A majority of the beers had a slight banana flavor, while pear was clearly perceived in many beers and was strongest in the center point composite sample (CS) and in sample Run 1. A cool mint sweet flavor was detected primarily in beers from Runs 9, 11, 12, and 16 where a direct correlation with the OAV of isoamyl acetate could be observed. In addition, a direct correlation between the OAV of isoamyl acetate and the flavor attributes banana and pear perceived by the tasters was shown. A honey flavor was also recognized in these samples, however, only to a weak degree. It could not be assigned to any secondary metabolite investigated in the PCA. (*E*)-*β*-damascenone, 2-phenylethyl acetate, and isobutyl acetate could be responsible for the slightly honey-like character based on the results of the investigations [41,42,48,49,50,51]. Even though the three substances could not be clearly identified as honey-like by olfactometry and showed no correlation in the PCA, an additive or synergistic effect might be possible [52,53]. Furthermore, phenylacetaldehyde is known for its honey-like flavor, however, it was not analyzed in this study [54].

According to the PCA, there was a direct correlation between the apple and berry flavors, which were perceived primarily in the beers from Runs 4, 6, 13, and 18, occurring in conjunction with the berry flavor being much stronger than the apple flavor. Beers from Runs 2, 5, 14, 15, and 17 were also dominated by a light apple and stronger berry flavor. A direct correlation is visible between apple and berry flavor with the OAV of (*E*)-*β*-damascenone. The results from GC-O associated with PCA suggest that the berry and apple flavors can indeed be attributed to the metabolite (*E*)-*β*-damascenone. The influence of acetaldehyde was negligible although the OAVs from Table 8 might have suggested an effect. In this case, it must be considered that the orthonasal odor threshold concentrations are related to individual flavor compounds in water, while the tasters perceived the complex flavor interplay in the beers. It has already been discussed that the acetaldehyde concentrations were far below the flavor threshold concentration in beer, and there is also the possibility that it was masked by other flavor substances.

### 3.3. Optimization Using RSM

Based on the analyses of ethanol, secondary metabolites, and sensory evaluation, the aim was to produce a beer with optimized properties. Since the focus was on the production of a non-alcoholic beer, the optimized beer should have the lowest possible ethanol content. At the same time, the DLG sum and the fruitiness of the beer should be maximized. Since isoamyl acetate with the flavor attributes of banana and pear is decisive for a fruity flavor, the aim was to maximize the concentration of this ester as well as the sum of esters. As (*E*)-*β*-damascenone had a direct correlation to the flavor characteristics apple and berry, its concentration should also be maximized as far as possible. In contrast, the aim was to minimize isovaleric acid and diacetyl concentrations to avoid undesirable off-flavors. To meet these specifications with a desirability of 66.5%, the optimization would reach the predicted interval shown in Table 9 at a fermentation temperature of 16.1 °C, an original gravity of 10.5 °P and a pitching rate of 5 × 10^6^ cells/mL.

Although asterisk labelled responses showed a significant LOF, in case of (*E*)-*β*-damascenone, ethanol, and sum DLG, the observed mean was within the predicted interval (PI). Only the isoamyl acetate concentration was almost three times higher than the predicted mean. However, since the aim of the optimization was to maximize the isoamyl acetate concentration, this was achieved by means of the selected fermentation parameters. The same applies to the fruitiness. Although the sum of esters showed an insignificant LOF, the analyzed value in the application case was almost twice as high as the predicted mean, so that the objective was also exceeded here. Nevertheless, the desired maximization was reached. The PI was not met for isovaleric acid. Still, since the specified target was to minimize the concentration, this target was exceeded while diacetyl stayed within the PI. Consequently, the optimized fermentation parameters, a moderate fermentation temperature, a low pitching rate, and—related to non-alcoholic beers—relatively high original gravity, delivered the desired results and even exceeded them in a positive way. Based on the desirability of 66.5%, it was expected that the predicted means would not be fully met within a 95% confidence interval. In fact, 50% of the values were within the predicted confidence interval, with the other values positively exceeding the prediction. In the supplementary material Table S4 and Figure S3, the tasting results of the predetermined flavor attributes of the optimized beer (mean values of triplicates) can be found including a spiderweb model showing the flavor profile. Compared to the beers Runs 1–20, in the optimized beer, the banana flavor in particular was much more noticeable and supported the strong fruitiness. This finding from the tasting is consistent with the high concentration of isoamyl acetate.

## 4. Discussion

As this study focused on (*E*)-*β*-damascenone, the associated findings and their implications are discussed below in the broadest context possible. The investigations of this study revealed that the flavor substance (*E*)-*β*-damascenone correlated with the apple and red berry flavor and, thus, contributed significantly to these flavor characteristics. Although (*E*)-*β*-damascenone is a well-known secondary metabolite in beer, and this substance is associated with apple or cooked apple- and berry-like flavors, it has not yet been reported as a main flavor component in non-alcoholic beers that contributes to berry flavors. A useful process proved to be first qualitatively analyzing which flavor component was causing the apple- and berry-like character in the beers using GC-O and subsequently quantitating the identified flavor substance (*E*)-*β*-damascenone by SIDA using a GC × GC-MS system. Although (*E*)-*β*-damascenone is predominantly associated with the apple or cooked apple flavor impression in the literature, further sources describe the flavor substance as fruity, peach-, rose-, and honey-like [12,13,15,47,48]. In general, (*E*)-*β*-damascenone is found naturally in various fruits [12,55]. The applied PCA clearly showed that the apple-like flavor detected during the sensory evaluations in this study could be associated with the presence of (*E*)-*β*-damascenone. Although it was already described in the introduction that numerous volatile substances are known to be responsible for apple flavor, no other correlations except for (*E*)-*β*-damascenone could be detected.

The fact that (*E*)-*β*-damascenone was perceived even more distinctively as a red berry flavor by the sensory assessors was also in agreement with existing literature. Gijs et al. described the flavor compound as red fruits, strawberry, and rhubarb [21]. Moreover, Saison et al. designated the flavor of the substance as red fruits, with attributes such as coconut and tobacco mentioned as well. The fact that less pleasant flavor impressions are partly associated with (*E*)-*β*-damascenone could be due to the fact that this secondary metabolite is often related to the aging of beers and is, therefore, used as an aging indicator [12,19,20]. Nevertheless, this study illustrates that the compound can be perceived as very pleasant. In addition, studies confirm that although (*E*)-*β*-damascenone may be an aging indicator, due to this, it does not necessarily have a direct sensory impact on the deterioration of the beer during the aging process [12,55]. Moreover, the concentration of (*E*)-*β*-damascenone is, thereby, decisive for the perceived character [56].

Due to its low orthonasal odor threshold concentration in water (0.006 µg/kg), only small amounts are required to perceive an impact by (*E*)-*β*-damascenone on the overall flavor, which was already demonstrated in different types of beers [16,41,57]. A study by Chevance et al. [58] explained that in a series of fresh Belgian beers, only low levels of (*E*)-*β*-damascenone of 6–25 µg/kg were found, which increased up to 210 µg/kg during aging. High concentrations of 450 µg/kg also occurred in investigations of the wort; however, these were reduced in the course of fermentation. The same phenomenon occurred not only in brewer’s wort, but could also be detected in a papaya juice substrate. Interestingly, different *C. saturnus* yeast strains reduced (*E*)-*β*-damascenone from papaya juice to trace levels during fermentation [13]. During this study, the opposite occurred. While ≤0.1 µg/L (*E*)-*β*-damascenone could be detected in the cooked brewer’s wort (100 °C for 5 min), the concentration range in the beers was between 0.451 and 0.571 µg/L. Thus, the concentration of (*E*)-*β*-damascenone did not decrease during the fermentation process, but increased instead. The fact that (*E*)-*β*-damascenone can also be formed during fermentation was also described in a wine study by Lloyd et al. [59], in the context of which six different grape musts were examined for this secondary metabolite. Without exception, the concentration in each sample increased over the course of fermentation, demonstrating that (*E*)-*β*-damascenone occurred via a sequential two-step process that required not only acidic conditions, but also yeast activity. Although the formation of (*E*)-*β*-damascenone by acid hydrolysis cannot be excluded in the context of this study, it is possible that the yeast metabolism of *C. saturnus* could also be responsible for its formation. However, the exact pathway of (*E*)-*β*-damascenone formation during fermentation cannot be explained so further research would need to be conducted in the future. In the literature, different pathways are described regarding the formation of the secondary metabolites [15]. It was reported that (*E*)-*β*-damascenone can be formed by oxidative cleavage from the carotenoid neoxanthin or from acid hydrolysis of plant secondary metabolites/glycosides during beer aging [48,55,58,60]. During wort boiling, it was observed that (*E*)-*β*-damascenone was mainly formed by the acid hydrolysis of glycoside precursors [21], though studies by Chevance et al. showed that the acid-catalyzed hydrolysis of glycosides in beer hardly occurred at a pH above 4.2 [58]. Nevertheless, it is likely that, in our study, the increasing (*E*)-*β*-damascenone formation in the brewer’s wort with increasing thermal input is due to acid hydrolysis. In terms of the (*E*)-*β*-damascenone formation during fermentation in our study, it is questionable whether acid-catalyzed hydrolysis of glycosides occurred. Since the pH values of the non-alcoholic beers were above 4.2 without exception (the lowest value was 4.74, cf. Table 4), this pathway is less likely based on the literature. Nevertheless, an oxidative reaction that may occur during beer aging is also questionable due to the freshness of the beers. Therefore, it can be assumed that yeast activity may have played a role in the formation of (*E*)-*β*-damascenone.

## 5. Conclusions

The main objective of this study was achieved, namely, to find out which volatile components the red berry as well as the apple flavor of the non-alcoholic beers produced with the yeast strains *C. saturnus* TUM 247 can be attributed to and which volatile substances were responsible for the overall fruity flavor. (*E*)-*β*-damascenone turned out to be a main flavor component of the red berry and apple flavor. The general fruitiness, besides (*E*)-*β*-damascenone, was mainly determined by isoamyl acetate, which was clearly assigned to the flavor characteristics pear, banana, and cool mint sweets. The influence of different fermentation parameters could be investigated and successfully optimized within the framework of response surface methodology by setting a maximum fruitiness without perceptible off-flavors during sensory evaluations. In addition, the aim of achieving the lowest possible ethanol content was reached. It should be emphasized with regard to yeast cell metabolism that *C. saturnus* TUM 247 can produce the highest ester quantity at a low pitching rate of 5 × 10^6^ cells/mL, almost regardless of the original gravity and at a moderate fermentation temperature. If the pitching rate increased, the sum of esters in the beers decreased, which is in contrast to the behavior often observed for traditional brewer’s yeasts.

## Figures and Tables

**Table 1:** Experimental design of response surface methodology (RSM) with face-centered, three-factorial (temperature, original gravity, and pitching rate) central composite design, and six replications of the center point (20 °C, 9 °P, 15 × 10^6^ cells/mL).

Run	Factor 1 A: Temperature (°C)	Factor 2 B: Original Gravity (°P)	Factor 3 C: Pitching Rate (×10^6^ cells/mL)
1	20	7	15
2	28	9	15
3 *	20	9	15
4	12	7	25
5	12	9	15
6	20	11	15
7 *	20	9	15
8 *	20	9	15
9	28	7	5
10 *	20	9	15
11	20	9	25
12	12	7	5
13	28	11	25
14	12	11	5
15	12	11	25
16	20	9	5
17	28	11	5
18	28	7	25
19 *	20	9	15
20 *	20	9	15

* Center points.

**Table 2:** Analytical standard methods of the wort and the beers according to MEBAK ^1^.

Analysis	Method	Device
Original gravity, ethanol content, apparent attenuation	MEBAK WBBM 2.9.6.3	Bending vibration and NIR spectroscopy, Alcolyzer Plus with DMA 5000 X sample 122 (Anton-Paar GmbH, Ostfildern, Germany)
pH value	MEBAK WBBM 2.13	pH meter with pH electrode, ProfiLine pH3210 pH meter (Xylem Inc., New York, NY, USA)
(Methylsulfanyl) methane (dimethyl sulfide)	MEBAK WBBM 2.23.1.1	GC-FID Clarus 580 (Perkin Elmer, Waltham, MA, USA), Column: 50 m × 0.32 mm Phenomenex FFAP, 0.25 μm
Fatty acid esters, fatty acids, 2-phenylethan-1-ol	MEBAK WBBM 2.23.6	GC-FID Clarus 580 (Perkin Elmer, Waltham, MA, USA), Column: 50 m × 0.32 mm Phenomenex FFAP, 0.25 μm
Acetaldehyde, ethyl acetate, isoamyl acetate, higher alcohols (propan-1-ol (n-propanol), 2-methylpropan-1-ol (isobutanol), amyl alcohols), ethyl methanoate (ethyl formate), ethyl propanoate (ethyl propionate)	MEBAK WBBM 2.21.1	GC-FID Clarus 580, Turbo Matrix 40, Head Space (Perkin Elmer, Waltham, MA, USA), Column: INNOWAX cross-linked polyethylene glycol, 60 m × 0.32 mm × 0.5 μm
Diacetyl, pentane-2,3-dione	MEBAK WBBM 2.21.5.1	GC-FID Clarus 580, Turbo Matrix 40, Head Space (Perkin Elmer, Waltham, MA, USA), Column: INNOWAX cross-linked polyethylene glycol, 60 m × 0.32 mm × 0.5 μm

^1^ MEBAK^®^ (2012), Editor: Dr. F. Jacob: The MEBAK collection of brewing analysis methods: Wort, beer and beer-based beverages. Collection of methods of the Mitteleuropäischen Brauchtechnischen Analysenkommission. Self-published by MEBAK.

**Table 3:** Analysis of variance (ANOVA) of selected responses using the three factors A: Temperature (°C), B: Original Gravity (°P), C: Pitching Rate (×10^6^ cells/mL) for response surface methodology (RSM).

Response	Unit	Minimum	Maximum	Model	*p*-Value	LOF *p*-Value
∑ Esters	mg/L	2.1	3.6	Quadratic	0.0012	0.1928
Isoamyl acetate	mg/L	0.7	1.9	Linear	0.0066	0.0065 *
Isovaleric acid	mg/L	0.53	1.30	Quadratic	<0.0001	0.8221
Diacetyl	mg/L	0.06	0.19	Linear	0.0005	0.4825
(*E*)-*β*-damascenone	µg/L	0.873	1.57	Linear	0.0058	<0.0001 *
Ethanol	% (*v*/*v*)	0.11	0.63	2FI ^1^	<0.0001	0.0210 *
Fruitiness	%	50	77	Linear	<0.0001	<0.0001 *
Sum DLG	points	3.93	4.70	Quadratic	0.0002	<0.0001 *

* Lack of fit (LOF) significant; target: LOF *p*-value > 0.10 insignificant; *p*-value < 0.05: significant. ^1^ 2FI = two-factor interaction.

**Table 4:** Analyzed values of original gravity (°P), ethanol (% *v*/*v*), apparent attenuation (%), and pH value in the 20 beers fermented with parameters of the response surface methodology (RSM) design.

Run	Original Gravity (°P)	Ethanol (% *v*/*v*)	Apparent Attenuation (%)	pH Value Beer
1	6.90	0.26	7.6	4.83
2	8.90	0.48	7.6	4.76
3 *	8.94	0.29	6.4	4.86
4	6.88	0.18	5.2	4.93
5	8.88	0.15	3.3	5.00
6	10.84	0.29	5.1	4.91
7 *	8.95	0.27	5.8	4.87
8 *	8.89	0.26	5.7	4.88
9	6.89	0.19	5.5	4.89
10 *	8.86	0.28	6.2	4.91
11	8.92	0.32	6.9	4.86
12	6.89	0.11	3.3	5.01
13	10.81	0.63	8.8	4.88
14	10.87	0.14	2.5	5.10
15	10.79	0.20	3.6	4.99
16	8.90	0.16	3.4	5.00
17	10.79	0.48	8.5	4.75
18	6.88	0.35	10.1	4.74
19 *	8.90	0.25	5.5	4.89
20 *	8.87	0.29	6.4	4.85

* Center points.

**Table 5:** Secondary metabolites analyzed by standard methods according to MEBAK ^1^ in the 20 beer samples fermented according to RSM design. Ethyl decanoate, ethyl formate, ethyl propionate, hexanoic acid, octanoic acid, decanoic acid, pentane-2,3-dione, 2-phenylethan-1-ol were measured additionally, however, are not shown as the concentrations stayed below the limit of quantitation (LOQ). An additional graphical representation can be found in the supplementary material Figure S1.

Run		1	2	3 *	4	5	6	7 *	8 *	9	10 *	11	12	13	14	15	16	17	18	19 *	20 *
Esters (mg/L)	Ethyl acetate	1.3	1.2	1.2	1.0	0.93	1.3	1.1	1.2	0.96	1.3	1.2	0.83	1.5	0.79	1.1	1.3	1.0	1.1	1.1	1.2
	Isoamyl acetate	1.4	0.8	1.1	0.8	0.7	1.0	1.0	1.2	1.9	1.2	1.3	1.3	0.8	1.2	0.7	2.7	1.3	0.8	1.1	1.1
	2-Phenylethyl acetate	0.73	0.36	0.62	0.50	0.38	0.62	0.65	0.59	0.67	0.57	0.70	0.58	0.39	0.49	0.42	1.1	0.43	0.45	0.62	0.81
	Isobutyl acetate	0.01	0.01	0.01	<LOQ	<LOQ	0.01	0.01	0.01	0.02	0.01	0.01	<LOQ	0.01	0.01	<LOQ	0.04	0.01	0.01	0.01	0.01
	Ethyl butanoate	<LOQ	0.01	0.01	0.01	0.01	0.01	0.01	0.01	0.01	0.01	0.01	0.01	0.01	0.01	0.01	0.01	0.01	0.01	0.01	0.01
	Ethyl hexanoate	0.02	0.02	0.02	0.03	0.03	0.02	0.02	0.01	0.03	0.02	0.02	0.03	0.01	0.03	0.03	0.02	0.02	0.02	0.02	0.02
	Ethyl octanoate	<LOQ	<LOQ	<LOQ	<LOQ	<LOQ	<LOQ	<LOQ	<LOQ	<LOQ	<LOQ	<LOQ	0.01	<LOQ	<LOQ	<LOQ	0.01	0.01	<LOQ	<LOQ	<LOQ
Organic acids (mg/L)	Isovaleric acid	1.2	1.2	1.1	0.76	0.61	1.1	1.1	1.1	0.74	1.0	1.1	0.54	1.2	0.53	0.62	0.83	0.86	1.2	1.1	1.3
Higher alcohols (mg/L)	n-Propanol	1.8	3.1	2.0	1.0	1.1	1.9	1.5	1.8	2.0	2.1	2.0	0.9	3.7	1.0	1.1	1.1	2.4	2.4	1.6	2.1
	Isobutyl alcohol	7.3	11.1	6.8	2.8	2.4	6.3	5.7	5.9	9.6	7.4	7.2	1.8	13.2	1.9	2.8	5.4	9.7	10.5	6.1	6.9
	Isoamyl alcohols	15.9	18.6	15.6	10.3	8.8	16.2	13.4	14.7	13.0	16.1	19.6	5.4	23.4	5.9	9.7	9.1	12.8	18.4	14.5	16.0
Aldehydes (mg/L)	Acetaldehyde	1.4	1.7	1.7	1.6	1.2	1.2	1.3	1.6	1.8	1.8	1.7	1.3	2.1	1.2	1.4	1.5	1.7	1.8	1.3	1.6
Ketones (mg/L)	Diacetyl	0.11	0.29	0.14	0.12	0.10	0.10	0.11	0.10	0.15	0.13	0.10	0.06	0.19	0.08	0.12	0.08	0.15	0.16	0.10	0.14
Sulfide (mg/L)	Dimethyl sulfide	0.016	0.022	0.024	0.015	0.023	0.028	0.022	0.021	0.017	0.022	0.024	0.020	0.029	0.029	0.030	0.021	0.031	0.019	0.023	0.022

* Center points; LOQ = Limit of quantitation; ^1^ MEBAK^®^ (2012), Editor: Dr. F. Jacob: The MEBAK collection of brewing analysis methods: Wort, beer, and beer-based beverages. Collection of methods of the Mitteleuropäischen Brauchtechnischen Analysenkommission. Self-published by MEBAK.

**Table 6:** Concentrations of (*E*)-*β*-damascenone (µg/L) in the beer samples fermented according to response surface methodology (RSM) design.

Run	Conc. (*E*)-*β*-Damascenone ^1^ (μg/L)	Range (*E*)-*β*-Damascenone (μg/L)
1	0.873	0.787–0.941
2	1.01	0.967–1.06
3 *	1.43	1.37–1.48
4	1.13	1.09–1.18
5	1.27	1.21–1.34
6	1.54	1.50–1.59
9	0.978	0.943–1.04
11	1.46	1.35–1.52
12	0.999	0.934–1.03
13	1.07	0.971–1.13
14	1.25	1.17–1.34
15	1.57	1.53–1.61
16	1.29	1.25–1.33
17	1.15	1.07–1.22
18	1.05	1.01–1.08

* Center point; ^1^ Mean of triplicates; coefficients of variation ≤9%.

**Table 7:** Sensory evaluation results as sum DLG and fruitiness (%) of 15 beer samples with six center points summarized as composite sample (CS).

Run	Sum DLG	Fruitiness (%)
1	4.25	53
2	4.60	68
CS *	4.70	68
4	4.23	50
5	4.60	65
6	4.50	68
9	4.32	57
11	4.63	62
12	3.93	38
13	4.65	77
14	4.67	75
15	4.70	68
16	4.48	62
17	4.60	73
18	4.40	63

* CS (Composite sample).

**Table 8:** Odor activity values (OAV) of the flavor compounds (*E*)-*β*-damascenone, isoamyl acetate, ethyl hexanoate, ethyl octanoate, ethyl decanoate, 2-phenylethyl acetate, and acetaldehyde.

Run	OAV ^1^						
	(*E*)-*β*-Damascenone	Isoamyl Acetate	Ethyl Hexanoate	Ethyl Ocanoate	Ethyl Decanoate	2-Phenylethyl Acetate	Acetaldehyde
1	150	190	17	<1	<1	2.0	88
2	170	110	17	<1	<1	1.0	110
CS *	240	150	17	<1	<1	1.8	97
4	190	110	25	<1	<1	1.4	100
5	210	97	25	<1	<1	1.1	75
6	260	140	17	<1	<1	1.7	75
9	160	260	25	<1	<1	1.9	110
11	240	180	17	<1	<1	1.9	110
12	170	180	25	1.1	<1	1.6	81
13	180	110	8.3	<1	<1	1.1	130
14	210	170	25	<1	<1	1.4	75
15	260	97	25	<1	<1	1.2	88
16	220	380	17	1.1	<1	3.1	94
17	190	180	17	1.1	<1	1.2	110
18	180	110	17	<1	<1	1.3	110

* CS (Composite sample). ^1^ The OAVs are calculated as a ratio of the concentration to the orthonasal odor threshold concentration in water. The orthonasal odor threshold concentrations in water are for (*E*)-*β*-damascenone 0.006 µg/kg, isoamyl acetate 7.2 µg/kg, ethyl hexanoate 1.2 µg/kg, ethyl octanoate 8.7 µg/kg, 2-phenylethyl acetate 360 µg/kg, and acetaldehyde 16 µg/kg [16].

**Table 9:** Validation of response surface methodology (RSM) model of selected responses with a two-sided predicted interval (PI) of 95%. Values based on triplicates.

Response	Unit	Predicted Mean	Observed Mean	Standard Deviation	95% PI Low	95% PI High
∑ Esters	mg/L	3.2	6.1	0.18	2.73	3.57
Isoamyl acetate *	mg/L	1.2	3.8	0.22	0.88	1.59
Isovaleric acid	mg/L	0.73	0.53	0.08	0.56	0.90
Diacetyl	mg/L	0.09	0.08	0.02	0.06	0.12
(*E*)-*β*-damascenone *	µg/L	1.30	1.41	0.14	1.07	1.54
Ethanol *	% (*v*/*v*)	0.19	0.19	0.04	0.13	0.26
Fruitiness *	%	71	87	3.67	64	76
Sum DLG *	points	4.62	4.61	0.08	4.45	4.78

* Significant models with significant LOF (lack of fit).

## Data Availability

The data presented in this study are available on request from the corresponding author.

## Supplementary Materials

The following supporting information can be downloaded at: www.mdpi.com/article/10.3390/foods11071038/s1, Sequence S1: *Cyberlindnera saturnus* TUM 247 26S sequence data from sequencing with NL1 primer, Table S1: Static headspace (HS) conditions, Table S2: One-sample *t*-test results of selected analyzed values of the six center point beers (Runs 3, 7, 8, 10, 19 and 20) determined for response surface methodology, Table S3: Predetermined flavor attributes for the tasting of the 15 beer samples. Numbers indicate how many sensory assessors recognized the specific flavor in the corresponding beer (n = 12), Table S4 + Figure S3 (Spiderweb): Predetermined flavor attributes for the tasting of the optimized beer sample (mean values of triplicates). Numbers indicate how many sensory assessors recognized the specific flavor in the beer (n = 12). Figure S1: Additional graphical representation of secondary metabolites analyzed by standard methods according to MEBAK in the 20 beer samples fermented according to RSM design, Figure S2: Pearson’s correlation of selected responses (fruitiness and (*E*)-*β*-damascenone) and factors A: Temperature, B: Original Gravity and C: Pitching Rate of the RSM for the determination of linear correlations between two variables where 1 stands for a strong positive correlation, 0 for no correlation and −1 for a strong negative correlation.
