# Supplementary material for: Influence of Varying Fermentation Parameters of the Yeast Strain Cyberlindnera saturnus on the Concentrations of Selected Flavor Components in Non-Alcoholic Beer Focusing on (E)-β-Damascenone
Source: Foods. 2022 Apr 2;11(7):1038. doi: 10.3390/foods11071038 (PMC8997441; doi:10.3390/foods11071038)

Supplementary Material

Sequence S1. *Cyberlindnera saturnus* TUM 247 26S sequence data from sequencing with NL1 primer.

AATCGCAGCATCCTAGGCAAAGCCGCAGTCCTCGGTCCAGATAGGCAACATCAACAAGGGCTATAACAC
ACCACCCGAAGGCAGTGCCACGTTCCAATGTCATTATCTTACCACCCGAACCGATGCTGGCCCAGTGAA
ATGCGAGTGCACAACCCAAGAAGGGAAGATAATCACAAAACACCAAGTCTGATCTAATACCCTTCCCTT
TCAACAATTTCACGTACTTTTTCACTCTCTTTTCAAAGTTCTTTTCATCTTTCCATCACTGTACTTGTTCGCT
ATCGGTCTCTCGCCAATATTTAGCTTTAGATGGAATTTACCACCCACTTAGAGCTGCATTCCCAAACAACT
CGACTCGTCGATAGAAATCTACAAAGCACTGGACACCCCGCCAGACGGGATTCTCACCCTCTGTGACGT
CCTGTTCCAAGGAACATAGACAAGGGCCAGCACCAGAAAACTATCTTCAAATTACAACTCGGGCACCAA
AGGTACCAGATTTCAAATTTGAGCTTTTGCCGCTTCACTCGCCGTTACTGAGGCAATCCCTGTTGGTTTCTT
TTCCTCCGCTTATTGG

Table S1. Static headspace (HS) conditions.

Parameter	Value
Equilibration time (min)	30
Injection/oven temperature (°C)	80
Sample line temperature (°C)	150
Transfer line temperature (°C)	160
Shaking level	3
Pressurizing time (min)	2
Pressure equilibration (min)	0.1
Injection time (min)	0.5
Needle flush time (min)	2
Sample loop (mL)	1

Table S2. One-sample *t*-test results of selected analyzed values of the six center point beers (Runs 3, 7, 8, 10, 19 and 20) determined for response surface methodology.

**Analyzed value**	**Prob > |*t*|**
Ethanol	1.62787^{-7}
Apparent attenuation	2.31548^{-7}
pH value beer	1.77322^{-13}
Ethyl acetate	2.22626^{-7}
Isoamyl acetate	2.97241^{-7}
2-Phenylethyl acetate	8.99319^{-6}
Isobutyl acetate	0
Ethyl butanoate	0
Ethyl hexanoate	1.08052^{-4}
Isovaleric acid	1.12333^{-6}
n-Propanol	1.11478^{-5}
Isobutyl alcohol	2.43523^{-6}
Isoamyl alcohols	3.43619^{-7}
Acetaldehyde	8.93629^{-5}
Diacetyl	2.03508^{-5}
Dimethyl sulfide	4.53495^{-8}

Figure S1. Additional graphical representation of secondary metabolites analyzed by standard methods according to MEBAK[1] in the 20 beer samples fermented according to RSM design.

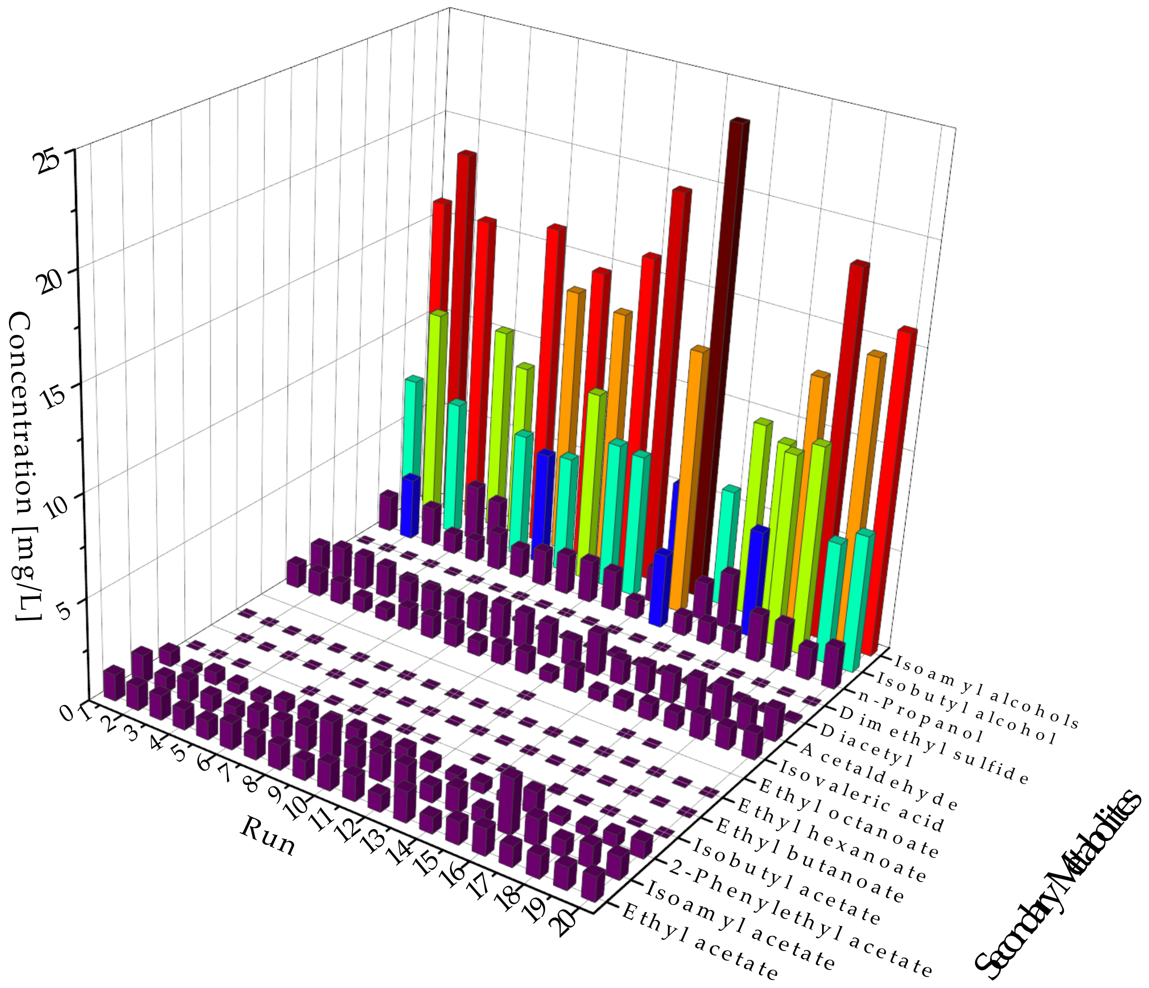

[1] MEBAK® (2012), Editor: Dr. F. Jacob: The MEBAK collection of brewing analysis methods: Wort, beer and beer-based beverages. Collection of methods of the Mitteleuropäischen Brauchtechnischen Analysenkommission. Self-published by MEBAK.

Figure S2. Pearson's correlation of selected responses (fruitiness and (*E*)-*β*-damascenone) and factors A: Temperature, B: Original Gravity and C: Pitching Rate of the RSM for the determination of linear correlations between two variables where 1 stands for a strong positive correlation, 0 for no correlation and –1 for a strong negative correlation.

	Run	A:Temperature	B:Original Gravity	C:Pitching Rate	Fruitiness	β-Damascenone
**Run**	1.000	0.110	0.258	-0.086	0.247	0.205
**A:Temperature**	0.110	1.000	0.000	0.000	0.333	-0.328
**B:Original Gravity**	0.258	0.000	1.000	0.000	0.799	0.529
**C:Pitching Rate**	-0.086	0.000	0.000	1.000	0.120	0.209
**Fruitiness**	0.247	0.333	0.799	0.120	1.000	0.455
**β-Damascenone**	0.205	-0.328	0.529	0.209	0.455	1.000

Table S3. Predetermined flavor attributes for the tasting of the 15 beer samples. Numbers indicate how many sensory assessors recognized the specific flavor in the corresponding beer (n = 12).

**Run**	**CS***	**1**	**2**	**4**	**5**	**6**	**9**	**11**	**12**	**13**	**14**	**15**	**16**	**17**	**18**
Apple	3	3	6	1	3	4	2	3	2	6	3	3	3	3	4
Banana	3	4	3	2	2	4	4	4	3	2	3	3	4	3	1
Red berry	2	3	5	6	5	5	3	2	2	8	5	3	0	5	7
Pear	8	9	6	4	5	4	4	7	5	4	5	4	7	7	3
Cool mint sweets	2	3	1	1	1	1	6	6	4	0	3	2	7	1	3
Solvent	0	0	0	1	0	0	1	0	3	0	0	0	1	1	1
Cheesy	0	0	0	2	0	0	0	0	0	0	0	1	0	0	2
Honey	4	2	2	0	2	4	4	4	4	4	0	0	0	4	0
Watery/pale	0	0	0	1	0	4	1	0	0	0	1	0	0	0	0

*CS (Composite sample) of six center points

Table S4 + Figure S3 (Spiderweb). Predetermined flavor attributes for the tasting of the optimized beer sample (mean values of triplicates). Numbers indicate how many sensory assessors recognized the specific flavor in the beer (n = 12).

Run	Optimized beer
Apple	4.3 ± 0.5
Banana	10.3 ± 0.5
Red berry	6.3 ± 0.5
Pear	7.7 ± 0.5
Cool mint sweets	8.7 ± 0.5
Solvent	1.0 ± 0.0
Cheesy	0
Honey	0
Watery/pale	0

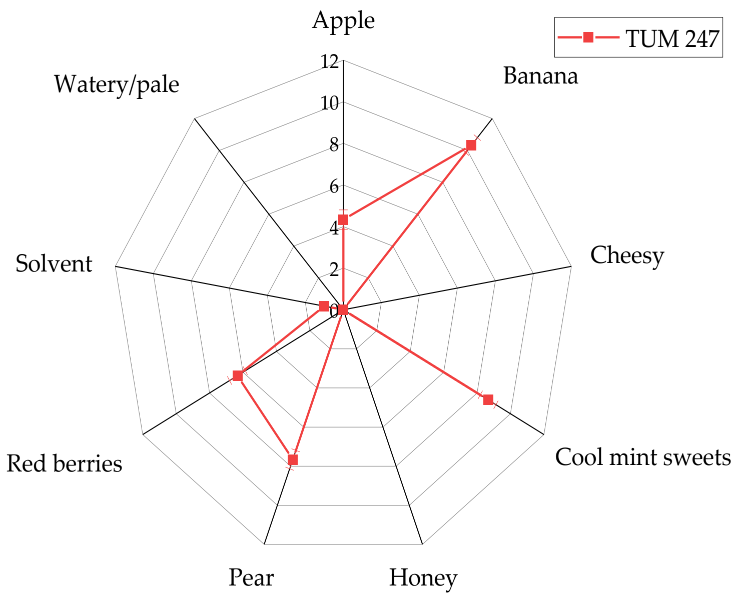

Supplement: Supplementary file 1 [file foods-11-01038-s001.zip › Foods_Supplementary material_final.pdf]
